# Activation of hepatic stellate cells by the ubiquitin C-terminal hydrolase 1 protein secreted from hepatitis C virus-infected hepatocytes

**DOI:** 10.1038/s41598-017-04259-7

**Published:** 2017-06-30

**Authors:** Ju-Chien Cheng, Ching-Ping Tseng, Mei-Huei Liao, Cheng-Yuan Peng, Jau-Song Yu, Po-Heng Chuang, Jing-Tang Huang, Jeremy J. W. Chen

**Affiliations:** 1 Department of Medical Laboratory Science and Biotechnology, China Medical University, Taichung, 40402 Taiwan; 2 Department of Medical Biotechnology and Laboratory Science, Chang Gung University, Taoyuan, 33302 Taiwan; 3 Graduate Institute of Biomedical Sciences, College of Medicine, Chang Gung University, Taoyuan, 33302 Taiwan; 4 Molecular Medicine Research Center, Chang Gung University, Taoyuan, 33302 Taiwan; 5 Department of Laboratory Medicine, Chang Gung Memorial Hospital, Taoyuan, 33302 Taiwan; 6 Institute of Biomedical Sciences, National Chung Hsing University, Taichung, 40402 Taiwan; 7 Department of Internal Medicine, China Medical University Hospital, Taichung, 40402 Taiwan; 8 Liver Research Center, Chang Gung Memorial Hospital, Linkou, 33302 Taiwan

## Abstract

Hepatitis C virus (HCV) infection of hepatocytes promotes liver fibrosis by activation of hepatic stellate cells (HSCs) and excessive deposition of extracellular matrix in liver tissue. Whether or not host factors released from the HCV-infected hepatocytes play role in HSCs activation is unclear. In this study, HSCs were activated by the conditioned medium derived from HCV replicon cells. Secretomic profiling of HCV replicon cells and the parental Huh7 cells revealed ubiquitin carboxy-terminal hydrolase L1 (UCHL1) as a novel secreted protein from HCV-infected hepatocytes. UCHL1 expression in hepatocytes was induced by HCV infection. UCHL1 was expressed in the liver and found in the plasma of patients with chronic hepatitis C. Molecular analysis by use of the anti-UCHL1 neutralization antibody and purified UCHL1 protein showed that secreted UCHL1 protein was bound to the cell surface of HSCs and activated JNK signaling leading to overexpression of alpha-smooth muscle actin and the activation of HSCs. These results provide further for understanding the underlying mechanism in HCV-mediated hepatic fibrogenesis.

## Introduction

More than 170 million people are infected with hepatitis C virus (HCV) worldwide^1^. Chronic hepatitis C (CHC) infection may result in liver damage that is characterized by liver fibrosis due to excessive deposition of extracellular matrix (ECM)^2, 3^. Repetitive HCV infection further causes fibrosing cholestatic hepatitis and promotes liver fibrosis to cirrhosis that frequently results in graft failure and death after transplantation^4, 5^. It is estimated that 20–30% of the patients with CHC infection progress to cirrhosis within 20 years post-infection^6^.

Hepatic fibrosis is a reversible process^7, 8^. Although direct-acting antiviral agents (DAA) with or without pegylated interferon (PEG-IFN) plus ribavirin is ineffective in the treatment of late stage HCV-induced liver fibrosis^9, 10^, the use of DAA in early stage liver fibrosis provides some improvement in patients with the added advantage of obtaining a positive health economic outcome^11^.

The activation of hepatic stellate cells (HSCs) is a key event in HCV-induced liver fibrosis^12^. HSCs are in the subendothelial space between hepatocytes and sinusoidal endothelial cells where they closely interact with hepatocytes and endothelial cells through numerous processes extending across the space of Disse^13^. A positive correlation between the number of activated HSCs and the stage of fibrosis is found in patients with CHC^14^. HSCs activation is usually accompanied by an increase in microfilaments which are mainly composed of alpha-smooth muscle actin (α-SMA). The expression of α-SMA is thereby a reliable marker for HSCs activation^15^.

HCV viral proteins activate HSCs^16^. HCV E2 protein induces pro-fibrogenic matrix metalloproteinase-2 expression that is involved in the degradation of normal liver ECM, an essential step in the progression of HCV-related hepatic fibrogenesis^17^. HCV core protein promotes HSCs proliferation, while the NS3 protease is pro-inflammatory by inducing transforming growth factor beta (TGF-β) signaling and collagen production in hepatic cells^18, 19^. Transgenic mice expressing the full-length HCV open reading frame in hepatocytes contributes to the development of hepatic fibrosis in the presence of carbon tetrachloride^20^. HCV subgenome replicon cells release TGF-β1 and other unidentified factors to induce procollagen gene expression in HSCs^21^. LX2 HSCs cultured in the conditioned medium (CM) from Huh7 cells stably expressing HCV core (Huh7-Core) induce high levels of α-SMA expression^22, 23^. These data imply that HCV induces secreted factors to activate HSCs *via* paracrine mechanisms, but the secreted factors have yet to be clearly identified.

Secretomics is a comprehensive method for identifying secreted proteins that are involved in a variety of biological regulatory processes^24^. In this study, secretome profiles of HCV replicon Con1 cells and parental Huh7 cells were compared and analyzed in order to define the host secreted proteins that play a role in HSCs activation.

## Results

### Conditioned medium from HCV replicon cells stimulated HSCs activation

**Figure 1:**
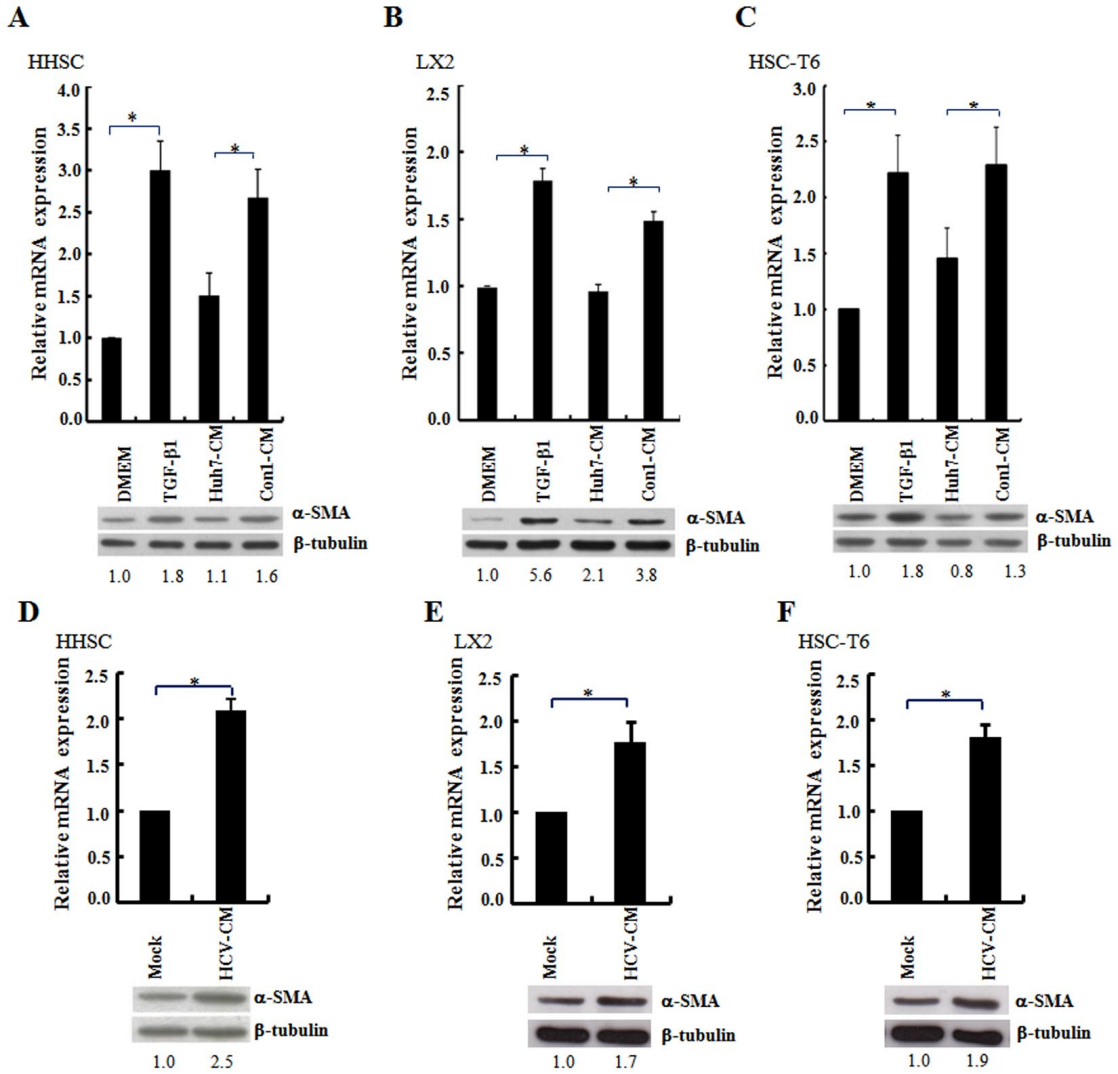
Conditioned medium from the culture of HCV replicon cells or HCV-infected cells increased procollagen I transcripts and α-SMA protein expression in HSC. (**A–F**) The HSCs of HHSC (panels A and D), LX2 (panels B and E) and HSC-T6 (panels C and F) were incubated with the conditioned medium (CM) derived from the 72 h culture of Huh7 and Con1 replicon cells (panels A,B and C), or the Huh7 cells infected with HCVcc (JFH1, MOI = 3; panels D,E and F). The level of procollagen I gene expression was quantified by real-time RT-PCR and the expression of β-tubulin was used as a control for normalization. Histograms were used to show the relative expression levels of procollagen I mRNA among different treatments. The cells cultured in DMEM without any treatment were arbitrarily denoted as 1. The data represent the mean ± S.D. (n = 3; *p < 0.05). In parallel, the cell lysates were harvested and Western blot analysis was performed using the anti α-SMA antibody. The ratios for the relative band intensities of α-SMA normalized by the expression of β-tubulin are shown at the bottom. HSCs cultured in the DMEM or DMEM supplemented with TGFβ1 (10 ng/ml) were used as the negative and positive control, respectively. The full Western blot and the corresponding positions of the molecular weight protein markers are presented in Supplementary Fig. S1.

Human (HHSC and LX2) and rat (HSC-T6) HSCs were grown in the conditioned medium collected from the culture of HCV Con1 replicon cells, HCVcc-infected cells, and the control Huh7 cells, respectively, to evaluate whether HCV infection of hepatocytes induces secretion of factors playing a role in HSCs activation. The conditioned medium from Con1 and HCVcc-infected cells and the HSCs activator TGF-β^25^ induced the expression of procollagen I transcript and α-SMA protein, the markers for HSCs activation and hepatic fibrosis^26^ in the three types of HSCs. The DMEM medium control and the conditioned medium from Huh7 cells had similar levels of procollagen 1 and α-SMA expression (Fig. 1A–F). These data imply that undefined secreted factors are present in the conditioned medium of HCV-infected hepatocytes that are able to induce HSC activation.

### Identification of proteins secreted from HCV Con1 replicon cells that induced HSC activation

**Figure 2:**
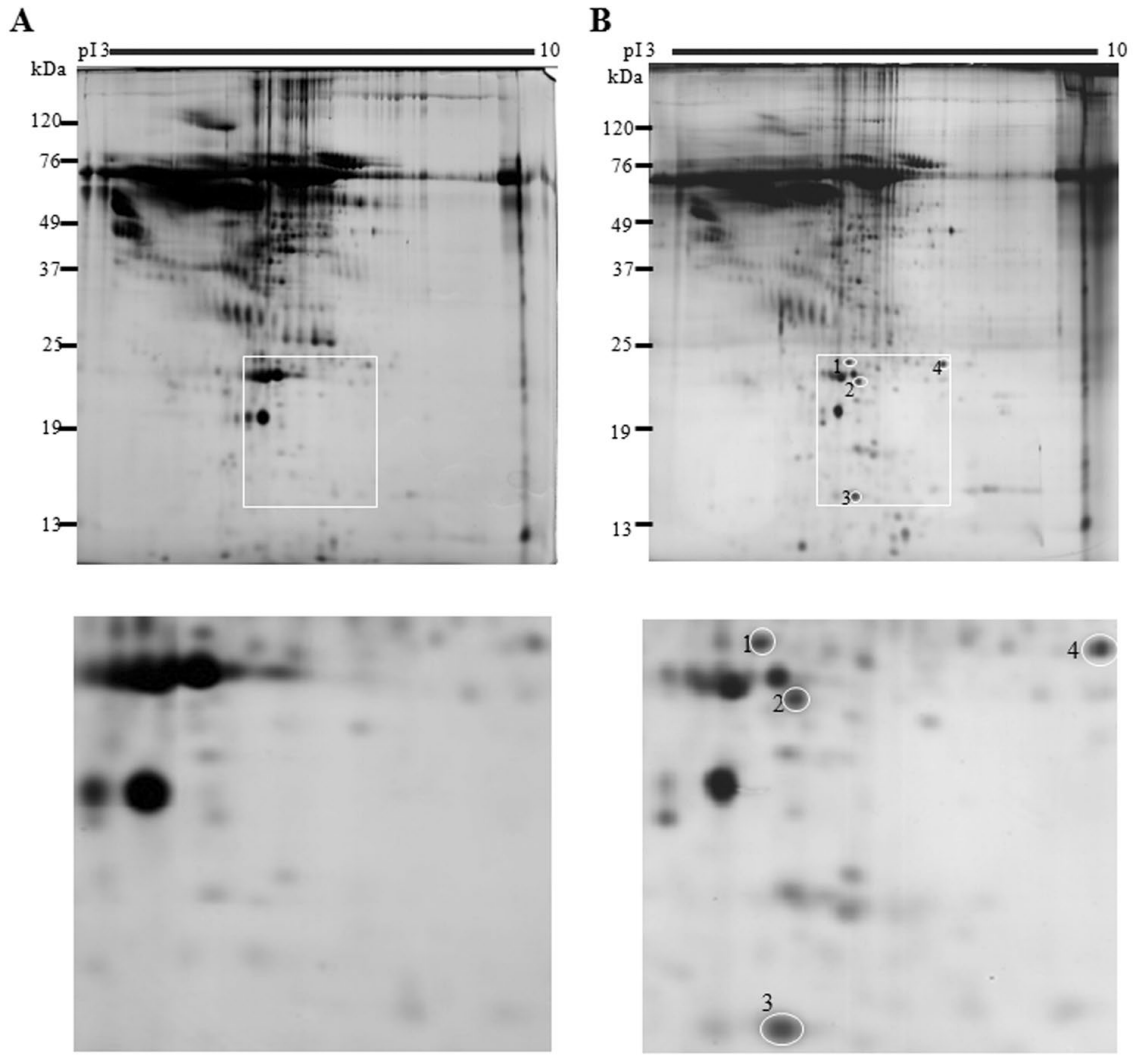
Secretome profiling of HCV replicon Con1 cell and Huh7 cells. (**A**,**B**) The conditioned medium derived from Huh7 (**A**) or Con1 (**B**) cells were collected as described in the Materials and Methods. The proteins that were present in the conditioned medium were separated by 2D-PAGE followed by silver staining of the proteins. The proteins that were differentially present in the conditioned medium from Huh7 and Con1 cells were labeled as 1, 2, 3, and 4 (upper and lower panels). The gel images for these four protein spots were enlarged as shown in the lower panel. These proteins were extracted from the gel and were identified by MALDI-TOF analysis.

The secretomes of the Con1 replicon cells and parental Huh7 cells were compared by two-dimensional polyacrylamide gel electrophoresis to identify the proteins that induce HSC activation. Four differentially expressed protein spots were present in the conditioned medium from HCV Con1 replicon cells, but not the parental Huh7 cells (Fig. 2A and B). These proteins were identified by MALDI-TOF analysis as UCHL1, GST-pi, TTR and TPI (Table 1).

**Table1:** Identification of candidate proteins by MALDI-TOF.

Spot number	Protein name	Gene symbol	Mass	pI	NCBI Accession #	Peptide matched	Sequence coverage (%)	MOWSE score
1	Ubiquitin carboxyl-terminal hydrolase L1	UCHL1	25.15	5.33	NP_004172	10	53	92
2	Glutathione S-transferase pi	GST Pi	23.56	5.43	NP_000843	11	48	75
3	Transthyretin	TTR	15.91	5.52	NP_000391	6	59	64
4	Triosephosphate isomerase 1	TPI1	26.9	6.45	NP_000356	14	75	129

**Figure 3:**
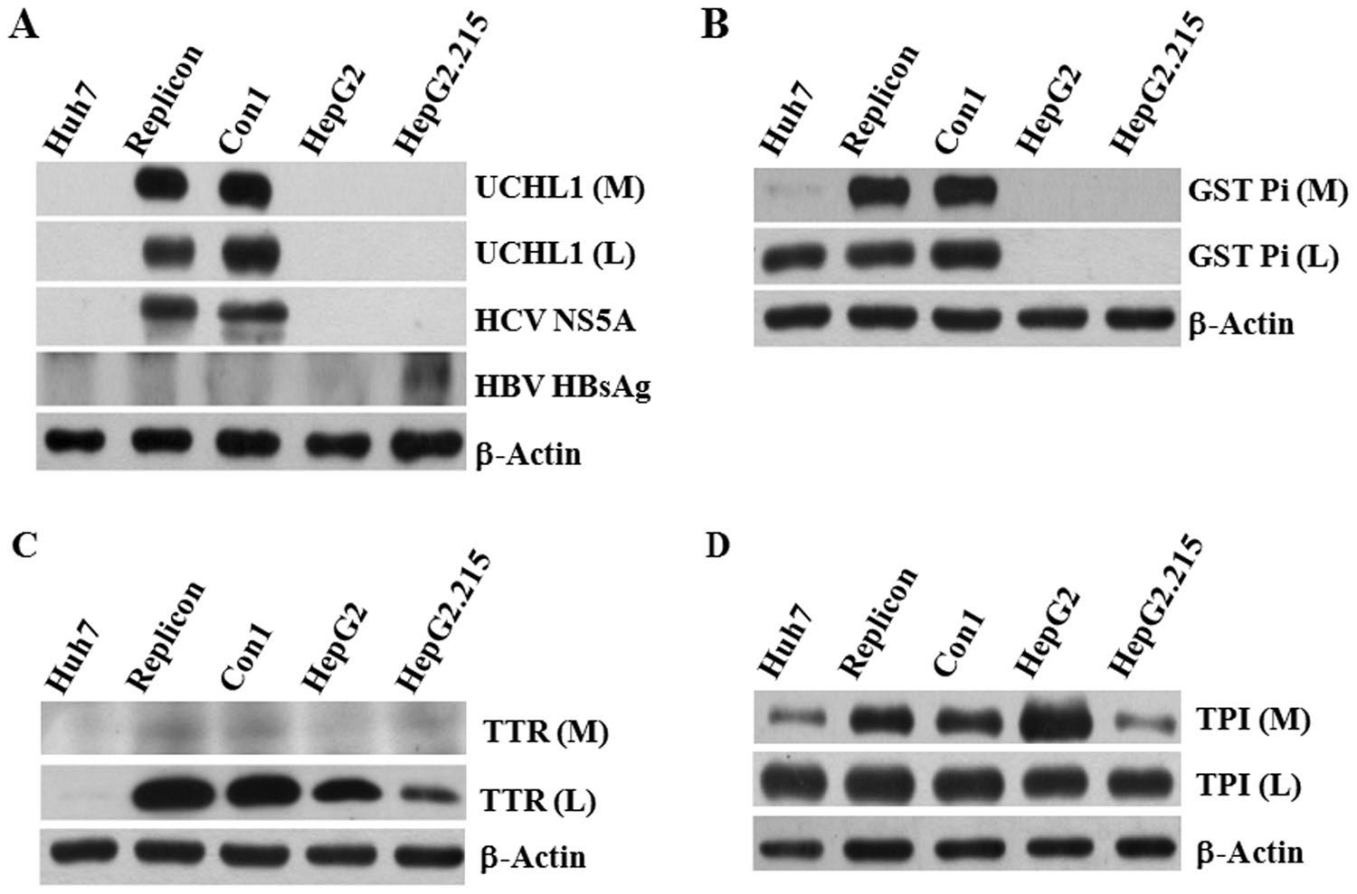
UCHL1 was specifically expressed in HCV replicon cells. (**A–D**) Fifty micrograms of protein from the cultured medium (M) or cell lysate (L) of the indicated cells were analyzed by Western blotting using the antibodies against UCHL1 (panel A), GSTpi (panel B), TTR (panel C) and TPI (panel D), respectively. HCV-NS5A and HBV-HB surface antigen (HBsAg) expression were used to confirm the authenticity of HCV and HBV genome-containing cell lines (panel A). The expression of β-actin in the cell lysates was used as a control for equal protein loading. The full Western blot and the corresponding positions of the molecular weight protein markers are presented in Supplementary Fig. S2.

Western blot analyses using the specific antibodies against the four proteins were performed to confirm their presence in the Con1 replicon cells and subgenomic replicon cells (Replicon), but not the parental Huh7 cells. UCHL1 was detected in the conditioned medium and cell lysates of both HCV replicon cells, but not the HepG2 and the HBV genome-containing cells HepG2.215 (Fig. 3A). UCHL1 was not detected in the conditioned medium from parental Huh7 cells (Fig. 3A). GST pi was present in the Huh7 cell lysate and was barely detectable in the conditioned medium of Huh7 (Fig. 3B). TTR was detected in the cell lysates of HCV subgenomic replicon cells, Con1, HepG2 and HepG2.215 but not Huh7. There was minimal expression of TTR in the conditioned medium from both HCV replicon cells and HepG2.215 (Fig. 3C). TPI was expressed in the cell lysates and conditioned medium of all tested cells. The conditioned medium from HCV replicon cells had significantly higher expression of TPI than the conditioned medium from Huh7 cells (Fig. 3D). These data indicate that among these proteins, UCHL1 is specifically expressed in HCV replicon cells and is released into the conditioned medium.

### Expression of UCHL1 in HCV-infected cells and in the liver hepatocytes and plasma of patients with CHC

**Figure 4:**
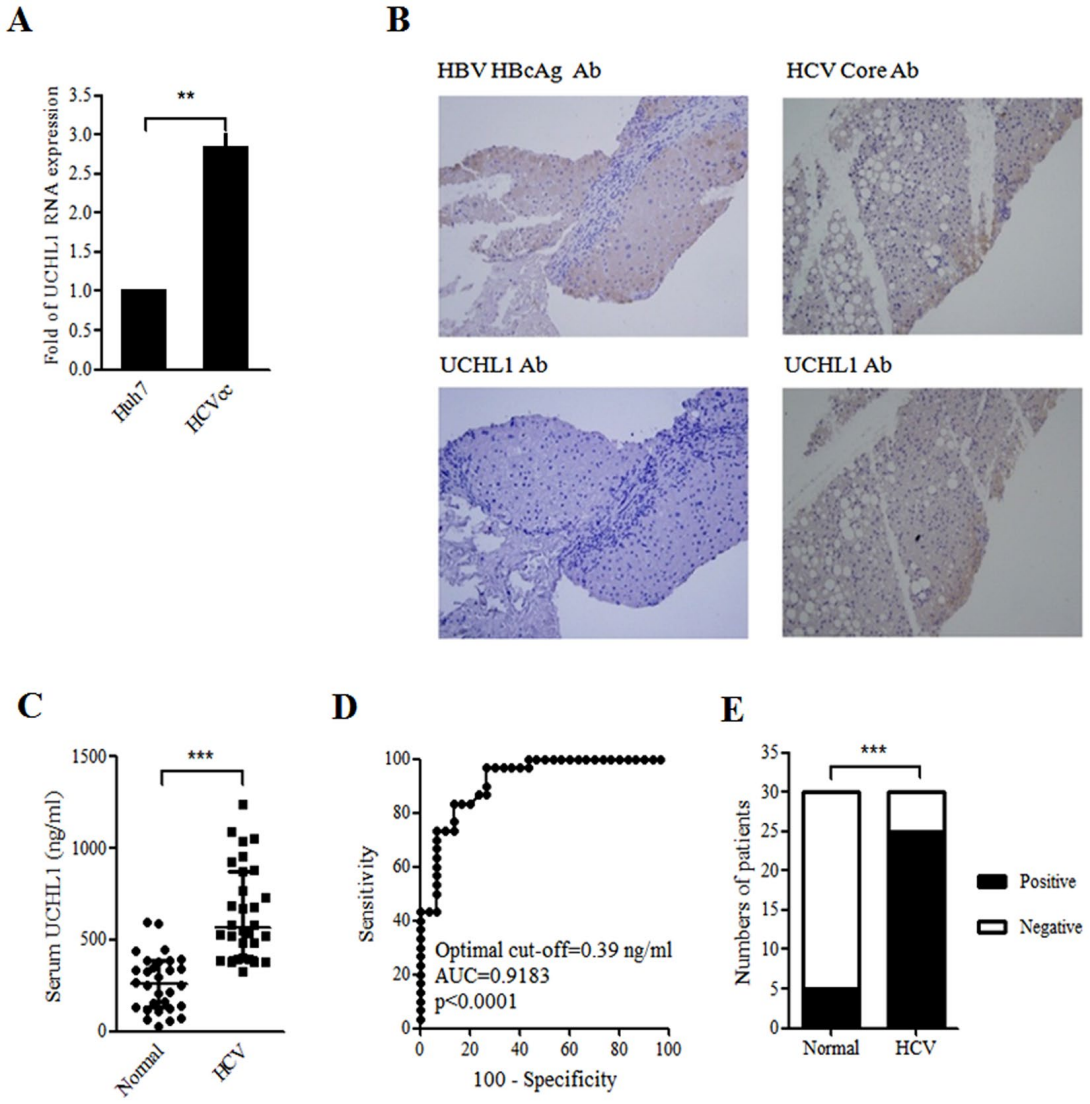
HCV induced UCHL1 expression in Huh7 cells and in the liver section and plasma of hepatitis C patients. (**A**) Huh7 cells were infected with HCVcc (JFH1, MOI 1) for 4 h and cultured for an additional 72 h after removing HCV infectious particles by washing three times with DMEM followed by suspension of the Huh7 cells in DMEM supplemented with 2% FBS. The cellular RNAs were collected for real-time RT-PCR analysis of UCHL1 mRNA expression. The data represent the mean ± S.D. of 3 independent experiments. **p < 0.01. (**B**) Liver biopsies from patients with CHC (right panel) and CBC (left panel) were subject to immunohistochemical staining of the indicated viral markers (upper panel) and UCHL1 (lower panel), respectively. Representative data are shown. The original magnification was 200X. (**C**) The expression levels of UCHL1 in the plasma of the healthy control and the patients with CHC were analyzed by ELSA assay. The plasma level of UCHL1 for each individual was shown in the scatter dot plot. The medium and the interquartile range for each group are indicated by the horizontal lines. ***p < 0.001. (**D**) ROC curve analysis was used to predict the clinical significance for the expression of plasma UCHL1. For the best compromise sensitivity/specificity, the area under the ROC curve was 0.9183 (95% CI: 0.8508 to 0.9859, p < 0.0001) with a sensitivity of 83.3% and a specificity of 86.7% for a cut-off value of 0.39 ng/ml. AUC, area under the curve; CI, confidence interval. (**E**) The individuals with plasma UCHL1 greater than 0.39 ng/ml were considered as positive for the UCHL1 test. The number for the healthy control and the patients with CHC who were positive and negative for the UCHL1 test are shown (n = 30). ***p < 0.001.

Cellular RNA from mock- and HCVcc-infected Huh7 cells were collected for measurement of UCHL1 expression to investigate whether UCHL1 is inducible by HCV infection. UCHL1 mRNA increased 2.8 fold in HCVcc-infected Huh7 cells when compared with control Huh7 cells (Fig. 4A), implying that UCHL1 expression is induced by HCV infection. Moderate increase in UCHL1 protein was also observed in Huh7 cells transiently infected with HCVcc (Supplementary Figure S5A).

The paraffin-embedded liver sections from patients with CHC (n = 5) and chronic hepatitis B (CHB, n = 5) were obtained and were analyzed by immunochemitry staining using anti-UCHL1 antibody to understand whether UCHL1 is specifically present in patients with CHC. UCHL1 was detected in the liver section from patients with CHC concomitant with the expression of HCV Core protein (Fig. 4B, right panel). HBcAg of HBV but not the UCHL1 was detected in the liver section from patients with CHB (Fig. 4B, left panel). This is consistent with the notion that UCHL1 is specifically induced by HCV infection.

Since UCHL1 was induced by HCV infection and secreted to the conditioned medium in the cell-based system, the following experiment was undertaken to establish whether or not UCHL1 is detectable in the plasma of patients with HCV infection. The plasma from patients with CHC (n = 30) and healthy donors (n = 30) was collected for quantifying the plasma levels of UCHL1 by ELISA. The medium levels of plasma UCHL1 was 261.2 ng/ml (interquartile range 131.8–383.9) and 647.8 ng/ml (interquartile range 401.8–875.3) for healthy donors and patients with CHC (p < 0.0001), respectively, as found by dot plot analysis (Fig. 4C). ROC analysis of the assay showed that the area under the curve (AUC) was 0.9183 (95% CI: 0.8508 to 0.9859, p < 0.0001) with a sensitivity of 83.3% and a specificity of 86.7% for a cut-off value of 0.39 ng/ml (Fig. 4D). 83% and 16.7% of patients with CHC and healthy individuals, respectively, had plasma levels of UCHL1 above the cut-off value, respectively (Fig. 4E, p < 0.001). The data indicate that the expression of UCHL1 is increased in liver fibrosis sections and the plasma of patients with CHC.

### UCHL1 mediated HCV-induced paracrine activation of HSCs

**Figure 5:**
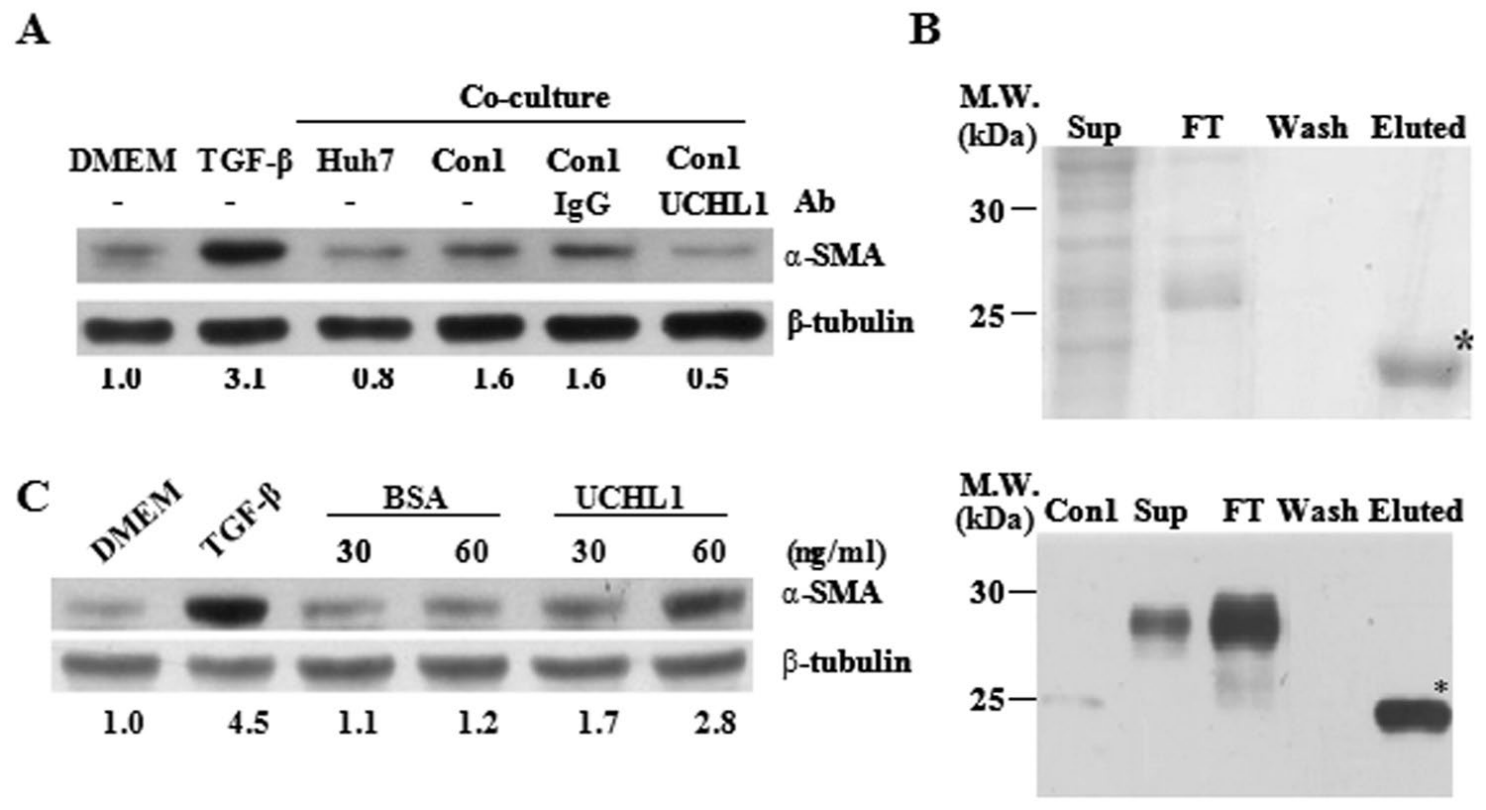
UCHL1 induced HSCs activation. (**A**) HSC-T6 cells (1 × 10^6^ cells) were co-cultured with Con1 or Huh7 cells (1 × 10^5^ cells) in the presence or absence of anti-UCHL1 antibody or mouse IgG control antibody for 24 h. The HSC-T6 cell lysate was collected and the expression of α-SMA was determined by Western blot analysis. HSC-T6 cells treated with DMEM and TGF-β1 were used as the negative and positive control of HSC activation, respectively. The expression of β-tubulin was used as the control for equal protein loading. The number below the gel images represents the relatively α-SMA expression levels for the indicated experimental condition after normalization with the expression of β-tubulin. (**B**) Recombinant UCHL1 protein fused to the Profinity eXact tag was expressed in *E*. *coli* BL21 after IPTG induction. Samples from the indicated purification steps were fractionated on a 12% SDS-PAGE and stained with Coomassie Brilliant Blue (upper panel). The protein with an expected molecular weight of UCHL1 (21 kDa) was noted in the eluted fraction after protease cleavage of the Profinity eXact tag and was indicated as *. The recombinant UCHL1 protein in the indicated steps of protein purification was further confirmed by Western blotting using the anti-UCHL1 antibody (lower panel). The cell lysate of Con1 was used as a positive control. (**C**) HSC-T6 cells were culture in DMEM w/o FBS for 24 h and then treated with the indicated concentrations of purified rUCHL1 or BSA for an additional 24 h. The cell lysates were collected and the expression of α-SMA was determined by Western blot analysis. The expression of β-tubulin was used as the control for equal protein loading. The number below the gel images represents the relatively α-SMA expression levels for the indicated experimental condition after normalization with the expression of β-tubulin. HSCs treated with DMEM and TGFβ1 (10 ng/ml) were used as negative and positive controls for HSC stimulation, respectively. The full Western blot and the corresponding positions of the molecular weight protein markers are presented in Supplementary Fig. S3.

Anti-UCHL1 neutralization antibody was added to the medium during co-culture of HSC-T6 cells with the HCV Con1 replicon cells to understand whether UCHL1 plays a role in HSC activation. HSC-T6 cells were activated by co-culture of Con1 but not Huh7 cells as indicated by an increase in the expression of α-SMA. The presence of UCHL1 neutralization antibody in the medium reduced the level of α-SMA expression when compared to treatment with control mouse IgG (Fig. 5A). Consistent with these observations, α-SMA expression in LX2 cells induced by the HCVcc-infected Huh7 cells conditioned medium was reduced in the presence of UCHL1 neutralized antibody (Supplementary Figure S5B). These data indicate that UCHL1 secreted into the medium induces paracrine activation of HSCs.

Recombinant UCHL1 (rUCHL1) protein (~28 kDa) was generated by using the pPAL7 expression vector and purified by the Profinity eXact™ protein purification systems to confirm UCHL1 is able to activate HSC. After removal of the Profinity eXact tag by protease cleavage, the eluted fraction contained an expected 21 kDa rUCHL1 protein that was specifically recognized by the anti-UCHL1 antibody (Fig. 5B). Treatment of different HSC cells with rUCHL1 proteins resulted in HSCs activation concomitant with a 2.2–3.6 fold increase in the expression of α-SMA protein when compared to the BSA-treated control (Fig. 5C and Supplementary S6A). UCHL1 thereby plays a role in HSC activation probably through paracrine stimulation.

### UCHL1 stimulated HSCs activation through JNK phosphorylation

**Figure 6:**
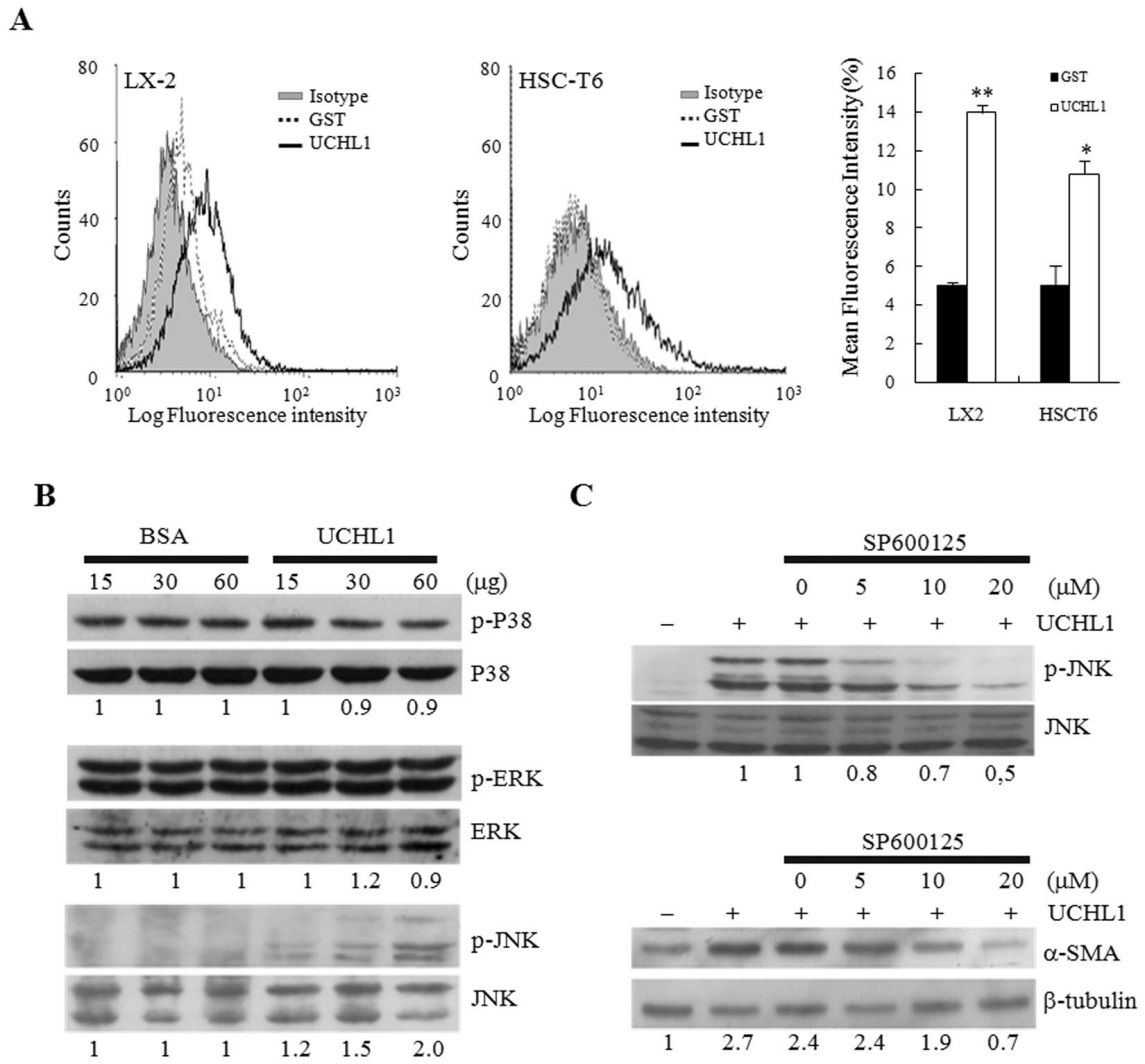
The underlying mechanism of UCHL1-induced HSC activation. (**A**) Purified UCHL1 or GST protein (60 μg) was preincubated with 1 × 10^6^ LX2 (left panel) or HSC-T6 (middle panel) cells, respectively. The binding of these proteins to the membrane surface of the cells was determined by incubation of anti-UCHL1, anti-GST, and isotype control antibodies, respectively, followed by incubation of FITC-conjugated goat anti-mouse secondary antibody and flow cytometric analysis. Purified GST protein was used as a control for non-specific binding. Isotype control IgG was used as the negative control. The mean fluorescence intensity for the indicated protein binding is shown in right panel. (**B**) HSC-T6 cells were treated with increasing concentrations of BSA or purified rUCHL1 protein for 24 h. The expression and the phosphorylation status of p38, ERK and JNK were analyzed by Western blotting. (**C**) HSC-T6 cells were treated with the indicated concentrations of the JNK inhibitor SP600125 for 30 min in DMEM supplemented with 0.2% FBS. Purified rUCHL1 (60 μg) protein was then added into the culture medium for 1 h to analyze the phosphorylation status of JNK and for 24 h to determine the expression of α-SMA. The expression of β-tubulin was used as the control for equal protein loading. The number below the gel images represents the relative α-SMA expression levels after normalization with the expression of β-tubulin. The full Western blot and the corresponding positions of the molecular weight protein markers are presented in Supplementary Fig. S4.

Whether or not UCHL1 binds directly to the cell surface of HSCs was determined by incubating rUCHL1 with the LX-2 and HSC-T6 cells followed by flow cytometry analysis using the anti-UCHL1 antibody and the fluorescein isothiocyanate (FITC)-labeled secondary antibody to identify the underlying mechanism of UCHL1-induced HSC activation. The binding of GST protein to cells was used as the control for non-specific binding. UCHL1, but not GST protein, bound to the cell surface of both LX-2 and HSC-T6 cells with an increase in the mean fluorescent intensity when compared to the antibody isotype control (Fig. 6A).

HSC-T6 cells were cultured in the medium in the presence or absence of purified rUCHL1 protein to define the signaling pathway underlying UCHL1-induced activation of HSCs. The phosphorylation status of the mitogen-activated protein kinase (MAP kinase) family members of ERKs, JNKs, and p38 that play critical roles in HSC activation^27, 28^ was analyzed by Western blotting at different time points after treatment of rUCHL1. JNK but not p38 or ERK phosphorylation was stimulated by UCHL1 (Fig. 6B). The JNK-specific inhibitor, SP600125, inhibited UCHL1-stimulated JNK phosphorylation and α-SMA expression in a dose-dependent manner (Fig. 6C). Similar results were observed in LX2 cells stimulated by rUCHL1 (Supplementary Figure S6B and C). UCHL1 thereby activates HSC through JNK phosphorylation and activation.

## Discussion

Liver fibrosis and the consequent cirrhosis and HCC are related to mortality of patients with CHC. Identification and characterization of the host factors that drive HSCs activation^29^ is of significance because the fibrosis is reversible. This will potentially result in developing clinically applicable biomarkers. HSCs account for 5–8% of total liver cells and are the major player in liver fibrosis. This is the first time that HCV-infected hepatocytes were shown to express and secrete UCHL1, which elicits paracrine stimulation of HSC by activating JNK signaling, providing an emerging point for monitoring liver fibrosis and interventional therapy for patients with CHC.

The HBe Ag and the HCV Core and NS3 are the major viral proteins playing a role in HSCs activation^18, 19, 30^. Host factors such as TGF-β and IL-13 are the predominant factors for HSC activation induced by HBV and HCV infection, respectively^31^, although the underlying mechanism remains largely unclear. UCHL1, a member of the ubiquitin carboxyl-terminal hydrolase family that catalyzes the hydrolysis of COOH-terminal ubiquityl esters and amides^32^, was defined as playing a role in HCV-induced HSCs activation. UCHL1 is a cytoplasmic deubiquitinating enzyme abundantly expressed in neurons and cells of the diffuse neuroendocrine system^32^. Impaired function of UCHL1 in mouse and human brain exhibits neuronal dysfunction that may be related to the development of neurodegenerative diseases, such as Parkinson’s and Alzheimer’s diseases^33, 34^. UCHL1 elicits oncogenic or tumor suppressive roles in different types of cancer^35, 36^, with low or silent UCHL1 expression in 77% of HCC cell lines^37^.

This study extends previous findings and demonstrates several unique features of UCHL1. First, UCHL1 expression was associated with HCV but not HBV infection. This is supported by the observations that UCHL1 was expressed in HCV replicon cells and HCV-infected cells, and in the liver fibrosis tissue section obtained from patients with CHC but not CHB. This is in accord with the observation that UCHL1 is up-regulated in the highly permissive HCV replicon cells^38^. Second, UCHL1 was not only a cytoplasmic protein but also a protein that is released from HCV-replicon and HCV-infected cells. UCHL1 is detectable in the culture medium of HCV-replicon and HCV-infected cells, and in the plasma of patients with CHC infection. UCHL1 is shown in the database of Vesiclepeida (Version 3.1) as present in the extracellular vehicles including exosomes, microvehicles and microparticles released by endothelial cells, B cells, colorectal and ovarian cancer cells, neuroblastoma, and in urine^39^. Finally, UCHL1 released by HCV-infected hepatocytes is sufficient to elicit paracrine activation of HSC. This is supported by the observations that ectopic expression of UCHL1 and the anti-UCHL1 antibody stimulates and blocks HSC activation, respectively. These findings highlight that HCV-induced expression of UCHL1 is a novel and specific mediator of HCV-related HSC activation and provide new insight into the molecular basis for the pathogenesis of HCV-induced HSCs activation and liver fibrosis.

Both viral and non-viral factors contribute to the development of liver fibrosis. HCV but not HBV was shown as of major viral-related etiology in inducing UCHL1 expression in infected hepatocytes and paracrine stimulation of HSC activation. UCHL1 also contributes to non-viral etiology in the activation of HSC. For example, UCHL1 is up-regulated in platelet-derived growth factor-activated HSC and is a regulator of HSC proliferation^40^. Concomitant with these findings, UCHL1 expression was positive in the liver sections of patients with HCV infection. It is also expressed in alcoholic liver disease, nonalcoholic steatohepatitis and primary biliary cirrhosis^40^. UCHL1 expression appears as a common molecular change associated with HCV infection and non-viral induced HSC activation and liver fibrosis.

How UCHL1 regulates HSCs activation was also addressed. JNKs and p38 signaling pathways have been reported to participate in the regulation of α-SMA expression during HSCs activation and differentiation^27, 41, 42^. JNK1 was further identified as an essential profibrogenic kinase in HSCs for the progression of liver fibrosis^43^. The secreted form of UCHL1 bound to an undefined cell surface receptor of HSCs and activated JNK but not the p38 signaling pathway, leading to an increase in α-SMA expression and HSC activation. This represents a new mode of action for UCHL1 in serving as an extracellular activator of JNK signaling. This is distinct from the intracellular UCHL1 in HSC that regulates the phosphorylation of retinoblastoma protein and the proliferative response of HSC^40^. Despite the fact that extracellular and intracellular UCHL1 elicit distinct molecular consequences, both lead to activation of HSC.

Liver fibrosis occurs at the early stage of HCV-induced severe liver diseases including HCC. Early detection and monitoring the progression of liver fibrosis allows rapid intervention to improve the outcome of anti-HCV treatment. Non-invasive abdominal examination by ultrasonic technology and transient elastography are the current clinical practice to screen disease progression of HCV-related liver disease^44^. The accuracy of image examination relies heavily on an experienced operator and cautious interpretation. Serum biomarkers provide an alternative approach to evaluate and monitor the progression of HCV-induced liver fibrosis for patients with CHC. Multiple serum biomarkers are usually used in combination. The FIBROSpect II test was designed for detecting the expression status for the proteins underlying the fibrogenic cascade, including hyaluronic acid, metallopeptidase inhibitor 1, and α2-macroglobulin^45^. The assay for detection of significant fibrosis has an AUC of 0.82–0.87 in the ROC analysis^46^. The current study revealed that UCHL1 is barely detectable in the plasma and liver tissue sections of individuals without HCV infection. In contrast, UCHL1 is highly expressed in the plasma of patients with CHC and in the liver tissue sections of patients with HCV-related fibrosis. The plasma level of UCHL1 clearly differentiates the individuals in the healthy control and patients with CHC, with the AUC of the ROC analysis reaching 0.9183. UCHL1 thereby may serve as an appropriate biomarker for monitoring the progression of CHC. A comprehensive cohort study is warranted to access the suitability of UCHL1 as a plasma biomarker for monitoring the progression of liver fibrosis in patients with an HCV infection.

This study demonstrated that UCHL1 expression is induced by HCV infection. UCHL1 stimulates JNK phosphorylation and signaling related to HSCs activation after binding to the cell surface of HSCs. The findings shed new light in understanding the underlying mechanisms of host factors in HCV pathogenesis and identified a potential diagnostic marker for HCV-related liver fibrosis and a potential novel anti-HCV adjuvant therapeutic approach.

## Methods

### Materials

The cell lines of HSC-T6 and LX2 were obtained from Dr. Scott L. Friedman (Mount Sinai School of Medicine, New York, NY). The human HSCs (HHSCs) were purchased from ScienCell Research Laboratories (Carlsbad, CA). The Huh7 cells were provided by Dr. Shih-Yen Lo (Tzu Chi University, Hualien, Taiwan). The Con1 replicon cells were kindly provided by Dr. Charles Rice (The Rockefeller University, New York, NY). The HepG2.215 cells and anti-HBV core antibody was provided by Dr. Shin-Lian Doong and Dr. Pei-Jer Chen (National Taiwan University, Taipei, Taiwan), respectively. The anti-HCV NS5A antibody was purchased from BioDesign (clone 388; Carmel, NY). The monoclonal anti-UCHL1 (clone 1B8-4D2) and anti-TT (clone 4D8TTR) antibodies were from Abnova (Taipei, Taiwan). The monoclonal anti-TPI antibody was purchased from Abcam (Cambridge, UK). The monoclonal anti-GSTpi antibody (clone 3/GST-π) was purchased from BD Biosciences (San Jose, CA). The monoclonal anti-β-tubulin antibody (Cat. No. GTX101279) was purchased from Upstate (Lake Placid, NY). The anti-α-SMA (clone 1A4) and anti-β-actin (clone AC-15) antibodies were purchased from Sigma (Saint Louis, Missouri). The monoclonal antibodies for p38, phospho-p38, ERK, phospho-ERK, JNK and phospho-JNK (Cat. No. #9212, #9211, #9102, #4370, #9252, and #9255) and the JNK inhibitor SP600125 were purchased from Cell Signaling Technology (Beverly, MA). The recombinant TGF-β1 protein was purchased from R&D Systems (Minneapolis, MN).

### Cell culture

The HSCT6, Huh7 and HepG2 cells were cultured in Dulbecco’s modified Eagle’s medium (DMEM) supplemented with 10% heat-inactivated fetal bovine serum (FBS). The LX2 cells were cultured in DMEM supplemented with 10% FBS and 1% glutamine. HHSCs were cultured in poly-L-lysine-coated (2 μg/cm^2^) plate using the Stellate Cell Medium (SteCM, Cat. No. 5301, ScienCell Research Laboratories) supplemented with 2% FBS. The Con1 and HepG2.215 cells were cultured in 10% FBS-supplemented DMEM in the presence of 800 μg/ml and 200 μg/ml G418, respectively.

### Generation of infectious HCV particles

Infectious HCV particles (HCVcc) were generated as described previously^47^. Briefly, the *in vitro* transcribed genomic JFH RNAs were delivered into Huh7.5 cells by electroporation. The HCVcc was recovered from cell culture medium after passage for 2 and 4 days. The viruses were collected and amplified as described previously^48^. The virus-containing supernatant was clarified by low-speed centrifugation, passed through a filter with the pore size of 0.45 μm, and concentrated by ultracentrifugation.

### Preparation of conditioned medium

For preparation of conditioned medium, Huh7 or Con1 cells were seeded at the density of 2 × 10^6^ cells/10 cm culture dish and cultured in DMEM supplemented with 10% FBS for 16 h. The cells were then washed with 1X phosphate buffered saline (PBS) twice and cultured in DMEM supplemented with 0.2% FBS for 72 h. Alternatively, Huh7 cells were infected with HCVcc (MOI = 3) for 4 h following incubation for another 72 h. The supernatant was collected and clarified by low-speed centrifugation and was considered as the conditioned medium for further assays.

### Co-culture system for HCV-induced HSC activation

HSCs (1 × 10^6^ cells) were seeded onto a 35 mm culture dish in DMEM supplemented with 10% FBS for 24 h. Starvation of HSCs was performed for another 24 h by culturing in DMEM in the absence of FBS. After changing the medium to DMEM supplemented with 0.2% FBS, an insert was placed into the culture dish for growing Con1 HCV replicon cells for 72 h. When performing the antibody blockage assay, 2 μg of antibody against UCHL1 and the respective control IgG were added into the insert when seeding the Con1 replicon cells. HSCs activation was monitored by Western blot analysis of the cell lysates for the expression of α-SMA.

### RNA isolation and real-time quantitative RT-PCR

Total cellular RNA was isolated by REzol C&T RNA extraction reagent as described by the manufacturer (Protech, Taipei). For quantification of procollagen I and UCHL1 mRNA expression, total cellular RNA (200 ng) was subject to one-step RT-PCR (20 μl) containing 2X SYBR Green PCR Master Mix and the primer set for procollagen I (procollagen-F: 5′-TGCGGAACCGGTGAGTACA-3′, and procollagen-R: 5′-CTTAAGGTTTAGGATTCGTGCTCAT-3′), and for UCHL1 (UCHL1-PF: 5′-TGGATGGCCACCTCTATGAAC-3′, and UCHL1-PR: 5′-CTTGCTCACGCTCGGTGA AT-3′). The reaction condition was 1 cycle of 48 °C for 30 min, 1 cycle of 95 °C for 10 min, and 40 cycles of 95 °C for 15 sec followed by 60 °C for 1 min using the ABI Prism 7000 Sequence Detection System. Distilled water was used to replace cDNA in each run as a negative control in PCR amplification. The relative gene expression was determined by the ΔΔCt method.

### Western blot analysis

The cell lysates were harvested and separated by 10% SDS-PAGE. The expression of the indicated proteins was detected using enhanced chemiluminescence kit (Pierce, Thermo Scientific) as described previously^47^.

### Two-dimensional polyacrylamide gel electrophoresis and silver staining

For protein separation at the first dimension, 300 μg of proteins were suspended in 300 μl of rehydration buffer (8 M urea, 2% CHAPS, 10 mg/ml dithiothreitol (DTT), and 0.5% Bio-Lyte) and applied to a ReadyStrip TM IPG Strip (pH 3–10, 17 cm; Bio-Rad) by the active rehydration method (50 V for 12 h). The rehydrated strips were focused in a four-step procedure: first ramped up to 250 V for 15 min, then linearly increased to 1,000 V for 3 h, maintained at 3,000 V until 10,000 V h in a total focusing at 20 °C, and finally decreased to 500 V for 12 h. Prior to the second dimension of protein separation, the focused strips were equilibrated in equilibration buffer (6 M urea, 2% sodium dodecyl sulfate (SDS), 0.375 M Tris-HCl, pH 8.8, 2% glycerol, and 20 mg/ml DTT) for 10 min twice, and reacted with the equilibration buffer supplemented with 2.5% iodoacetamide for 10 min. The equilibrated strips were positioned on a 12% polyacrylamide gel and protein separation was performed at a constant current (15 mA). Protein spots on the gel were visualized by silver staining and were identified by in-gel tryptic digestion and MALDI-TOF MS as previously described^49^.

### Clinical specimen

Liver biopsies and the peripheral blood of the patients with HCV or HBV infection were obtained from China Medical University Hospital with the Institutional Review Board approval ID of DMR94-IRB-172 and CMUH103-REC-068. All patients enrolled in this study have signed written informed consent.

### Immunohistochemistry

Immunohistochemistry (IHC) of liver biopsy tissue sections was performed as described previously^50^. Briefly, liver sections were deparaffinized in xylene and rehydrated through a series of graded ethanol. Antigen retrieval was performed by placing the tissue sections in a Tris-EDTA buffer containing 10 mM Tris-HCl (pH 9.0), 1 mM EDTA and 0.05% Tween 20, and heated to above 65 °C in a microwave for 10 min. The endogenous peroxidase was quenched by using 3% hydrogen peroxide for 10 min. Non-fat dried milk (5%) was applied to the sections at room temperature for 1 h to block the non-specific antibody binding sites. After incubating with the indicated antibodies overnight at 4 °C, the tissue sections were washed extensively using 1X PBS. The antigen-antibody complexes were then detected by using the Super Sensitive^TM^ Link-Label IHC Detection System (BioGenex, Fremont, CA) and the cell nucleus was counter-stained by hematoxylin for 30 sec. After another extensive wash, the sections were dehydrated through a graded ethanol series ending in water, mounted and observed by microscopy (Olympus BX50).

### Detection of plasma UCHL1 by ELISA

Plasma levels of UCHL1 were measured by using the ELISA Kit for UCHL1 according to the manufacturer’s protocols (USCN life science, Wuhan, China). Briefly, 100 μl of diluted samples and standards were placed into the reaction wells of a microplate for 2 h. After removing the solution, Detection Reagent A (100 μl) was added into the well for incubation at 37 °C for 1 h. The wells were then washed three times using the washing buffer followed by adding 100 μl of Detection Reagent B for incubation at 37 °C for 30 min. After several washes with the washing buffer, the substrate solution (90 μl) was added into the wells and incubated at 37 °C for 30 min. The reaction was terminated by adding 50 μl of stop solution and the absorbance was measured at 450 nm in a spectrophotometer. The plasma concentration of UCHL1 in the samples was then determined by comparing the O.D. of the samples to the standard curve.

### Expression and purification of UCHL1

To generate full-length UCHL1 cDNA, the total RNA from Con1 cells was reverse-transcribed by random hexamer followed by PCR amplification using the forward primer UCHL1-F (5′-aagcttATGCAGCTCAAGCCGATG G-3′) and reverse primer UCHL1-R (5′-ctcgaggTTAGGCTGCCTTGC-3′). The ~0.7 kb PCR product was cloned into pT&A vector (Yestern Biotec, Taipei, Taiwan) to generate pT&A-UCHL1. The *Hind* III-*Xho* I fragment of pT&A-UCHL1 containing the full-length UCHL1 cDNA was further subcloned into the *Hind* III-*Xho* I sites of pPAL7 expression vector (Bio-Rad, Richmond, CA) to generate pPAL7-UCHL1. For production and purification of recombinant UCHL1 protein (rUCHL1), the pPAL7-UCHL1 plasmid was transformed into *E*. *coli* BL21 (DE3) followed by induction of protein expression with 1 mM isopropyl-β-D-thiogalactopyranoside (IPTG) at 37 °C for 4 h. Subsequently, the bacterial suspension was centrifuged at 13,000 rpm at 4 °C for 15 min. The supernatant rUCHL1 protein was purified by using the Profinity eXact™ Protein Purification Systems (Bio-Rad, Richmond, CA) and was concentrated using an Amicon Ultra-4^®^ 10 K (Millipore, Bedford, MA) for further analysis.

### Flow cytometric assay for the binding activity of UCHL1 to HSCs

Purified rUCHL1 or GST proteins (60 μg) were preincubated with the indicated HSCs (1 × 10^6^ cells) for 1 h at room temperature. After centrifugation at 300 rpm and washing twice with 1X PBS, the cell pellets were blocked by 1% BSA for an additional 1 h at room temperature. After washing twice with 1X PBS, the anti-UCHL1, anti-GST and control anti-IgG antibodies were mixed with the cell pellet, respectively, and incubated at 4 °C for 1 h. After washing twice with 1X PBS, the Alexa Fluor-488 conjugated anti-mouse IgG was added to the cell-antibody mixture. The binding of UCHL1 to HSCs was measured by flow cytometric analysis (FACSCanto, BD).

### Statistical analysis

The GraphPad Prism version 5.0 was used for statistical analysis. Student’s t-test was used in comparison analysis with the nonparametric data being assessed using the Mann-Whitney test. P < 0.05 was considered as statistically significant.

## Electronic supplementary material

Supplementary Information file

## References

[1] Mohd Hanafiah K, Groeger J, Flaxman AD, Wiersma ST (2013). Global epidemiology of hepatitis C virus infection: new estimates of age-specific antibody to HCV seroprevalence. Hepatology.

[2] Poynard T, Yuen MF, Ratziu V, Lai CL (2003). Viral hepatitis C. Lancet.

[3] Hernandez-Gea V, Friedman SL (2011). Pathogenesis of liver fibrosis. Annual review of pathology.

[4] Berenguer M (2003). Severe recurrent hepatitis C after liver retransplantation for hepatitis C virus-related graft cirrhosis. Liver transplantation: official publication of the American Association for the Study of Liver Diseases and the International Liver Transplantation Society.

[5] Duarte-Rojo A (2013). Interleukin-28B and fibrosing cholestatic hepatitis in posttransplant hepatitis C: a case-control study and literature review. Liver transplantation: official publication of the American Association for the Study of Liver Diseases and the International Liver Transplantation Society.

[6] Schuppan D, Krebs A, Bauer M, Hahn EG (2003). Hepatitis C and liver fibrosis. Cell death and differentiation.

[7] Povero D (2010). Liver fibrosis: a dynamic and potentially reversible process. Histology and histopathology.

[8] Ellis EL, Mann DA (2012). Clinical evidence for the regression of liver fibrosis. Journal of hepatology.

[9] Hezode C (2013). Triple therapy in treatment-experienced patients with HCV-cirrhosis in a multicentre cohort of the French Early Access Programme (ANRS CO20-CUPIC) - NCT01514890. Journal of hepatology.

[10] Kattakuzhy S (2016). Moderate Sustained Virologic Response Rates With 6-Week Combination Directly Acting Anti-Hepatitis C Virus Therapy in Patients With Advanced Liver Disease. Clinical infectious diseases: an official publication of the Infectious Diseases Society of America.

[11] Younossi ZM (2015). Cost-effectiveness of all-oral ledipasvir/sofosbuvir regimens in patients with chronic hepatitis C virus genotype 1 infection. Alimentary pharmacology & therapeutics.

[12] Tsukada S, Parsons CJ, Rippe RA (2006). Mechanisms of liver fibrosis. Clinica chimica acta; international journal of clinical chemistry.

[13] Ueno T (1997). Hepatic stellate cells and intralobular innervation in human liver cirrhosis. Human pathology.

[14] Tomanovic NR (2009). Activated liver stellate cells in chronic viral C hepatitis: histopathological and immunohistochemical study. Journal of gastrointestinal and liver diseases: JGLD.

[15] Carpino G (2005). Alpha-SMA expression in hepatic stellate cells and quantitative analysis of hepatic fibrosis in cirrhosis and in recurrent chronic hepatitis after liver transplantation. Digestive and liver disease: official journal of the Italian Society of Gastroenterology and the Italian Association for the Study of the Liver.

[16] Mengshol JA, Golden-Mason L, Rosen HR (2007). Mechanisms of Disease: HCV-induced liver injury. Nature clinical practice. Gastroenterology & hepatology.

[17] Mazzocca A (2005). Binding of hepatitis C virus envelope protein E2 to CD81 up-regulates matrix metalloproteinase-2 in human hepatic stellate cells. The Journal of biological chemistry.

[18] Bataller R, Paik YH, Lindquist JN, Lemasters JJ, Brenner DA (2004). Hepatitis C virus core and nonstructural proteins induce fibrogenic effects in hepatic stellate cells. Gastroenterology.

[19] Sakata K (2013). HCV NS3 protease enhances liver fibrosis via binding to and activating TGF-beta type I receptor. Scientific reports.

[20] Chouteau P (2012). Hepatitis C virus (HCV) protein expression enhances hepatic fibrosis in HCV transgenic mice exposed to a fibrogenic agent. Journal of hepatology.

[21] Schulze-Krebs A (2005). Hepatitis C virus-replicating hepatocytes induce fibrogenic activation of hepatic stellate cells. Gastroenterology.

[22] Clement S (2010). The hepatitis C virus core protein indirectly induces alpha-smooth muscle actin expression in hepatic stellate cells via interleukin-8. Journal of hepatology.

[23] Coenen M (2011). Hepatitis C virus core protein induces fibrogenic actions of hepatic stellate cells via toll-like receptor 2. Laboratory investigation; a journal of technical methods and pathology.

[24] Zullo J, Matsumoto K, Xavier S, Ratliff B, Goligorsky MS (2015). The cell secretome, a mediator of cell-to-cell communication. Prostaglandins & other lipid mediators.

[25] Friedman SL (2008). Mechanisms of hepatic fibrogenesis. Gastroenterology.

[26] Nouchi T, Tanaka Y, Tsukada T, Sato C, Marumo F (1991). Appearance of alpha-smooth-muscle-actin-positive cells in hepatic fibrosis. Liver.

[27] Kluwe J (2010). Modulation of hepatic fibrosis by c-Jun-N-terminal kinase inhibition. Gastroenterology.

[28] Nakagawa H, Maeda S (2012). Molecular mechanisms of liver injury and hepatocarcinogenesis: focusing on the role of stress-activated MAPK. Pathology research international.

[29] Tacke F, Weiskirchen R (2012). Update on hepatic stellate cells: pathogenic role in liver fibrosis and novel isolation techniques. Expert review of gastroenterology & hepatology.

[30] Zan Y, Zhang Y, Tien P (2013). Hepatitis B virus e antigen induces activation of rat hepatic stellate cells. Biochemical and biophysical research communications.

[31] Weng HL (2009). The etiology of liver damage imparts cytokines transforming growth factor beta1 or interleukin-13 as driving forces in fibrogenesis. Hepatology.

[32] Wilkinson KD (1989). The neuron-specific protein PGP 9.5 is a ubiquitin carboxyl-terminal hydrolase. Science.

[33] Saigoh K (1999). Intragenic deletion in the gene encoding ubiquitin carboxy-terminal hydrolase in gad mice. Nature genetics.

[34] Setsuie R (2007). Dopaminergic neuronal loss in transgenic mice expressing the Parkinson’s disease-associated UCH-L1 I93M mutant. Neurochemistry international.

[35] Wulfanger, J. *et al*. Heterogeneous expression and functional relevance of the ubiquitin carboxyl-terminal hydrolase L1 in melanoma. *International journal of cancer* **133** (2013). 10.1002/ijc.28278 23686552

[36] Zheng S (2015). Heterogeneous expression and biological function of ubiquitin carboxy-terminal hydrolase-L1 in osteosarcoma. Cancer letters.

[37] Yu J (2008). Epigenetic identification of ubiquitin carboxyl-terminal hydrolase L1 as a functional tumor suppressor and biomarker for hepatocellular carcinoma and other digestive tumors. Hepatology.

[38] Ye F (2015). Quantitative Proteomics Analysis of the Hepatitis C Virus Replicon High-Permissive and Low-Permissive Cell Lines. PloS one.

[39] Lee HS, Jeong J, Lee KJ (2009). Characterization of vesicles secreted from insulinoma NIT-1 cells. Journal of proteome research.

[40] Wilson CL (2015). Ubiquitin C-terminal hydrolase 1: A novel functional marker for liver myofibroblasts and a therapeutic target in chronic liver disease. Journal of hepatology.

[41] Garat, C. *et al*. Induction of smooth muscle alpha-actin in vascular smooth muscle cells by arginine vasopressin is mediated by c-Jun amino-terminal kinases and p38 mitogen-activated protein kinase. *The Journal of biological chemistry* **275** (2000). 10.1074/jbc.M003000200 10807920

[42] Yoshida K (2005). Transforming growth factor-beta and platelet-derived growth factor signal via c-Jun N-terminal kinase-dependent Smad2/3 phosphorylation in rat hepatic stellate cells after acute liver injury. The American journal of pathology.

[43] Zhao G (2014). Jnk1 in murine hepatic stellate cells is a crucial mediator of liver fibrogenesis. Gut.

[44] Smith JO, Sterling RK (2009). Systematic review: non-invasive methods of fibrosis analysis in chronic hepatitis C. Alimentary pharmacology & therapeutics.

[45] Patel K (2008). Correlation of FIBROSpect II with histologic and morphometric evaluation of liver fibrosis in chronic hepatitis C. Clinical gastroenterology and hepatology: the official clinical practice journal of the American Gastroenterological Association.

[46] Valva P, Rios DA, De Matteo E, Preciado MV (2016). Chronic hepatitis C virus infection: Serum biomarkers in predicting liver damage. World journal of gastroenterology.

[47] Huang JT (2013). Hepatitis C virus replication is modulated by the interaction of nonstructural protein NS5B and fatty acid synthase. Journal of virology.

[48] Steenbergen RH (2013). Human serum leads to differentiation of human hepatoma cells, restoration of very-low-density lipoprotein secretion, and a 1000-fold increase in HCV Japanese fulminant hepatitis type 1 titers. Hepatology.

[49] Wu CC (2010). Candidate serological biomarkers for cancer identified from the secretomes of 23 cancer cell lines and the human protein atlas. Molecular & cellular proteomics: MCP.

[50] Zeremski M (2008). Intrahepatic levels of CXCR3-associated chemokines correlate with liver inflammation and fibrosis in chronic hepatitis C. Hepatology.

