# Supplementary material for: Activation of hepatic stellate cells by the ubiquitin C-terminal hydrolase 1 protein secreted from hepatitis C virus-infected hepatocytes
Source: Sci Rep. 2017 Jun 30;7:4448. doi: 10.1038/s41598-017-04259-7 (PMC5493679; doi:10.1038/s41598-017-04259-7)
Supplement: Supplementary file 1 — Supplementary Information file [file 41598_2017_4259_MOESM1_ESM.doc]

**Supplementary Information file**

**Activation of hepatic stellate cells by the ubiquitin C-terminal hydrolase 1 protein secreted from hepatitis C virus-infected hepatocytes**

Ju-Chien Cheng, Ching-Ping Tseng, Mei-Huei Liao, Cheng-Yuan Peng, Jau-Song Yu, Po-Heng Chuang, Jing-Tang Huang, Jeremy J.W. Chen

**
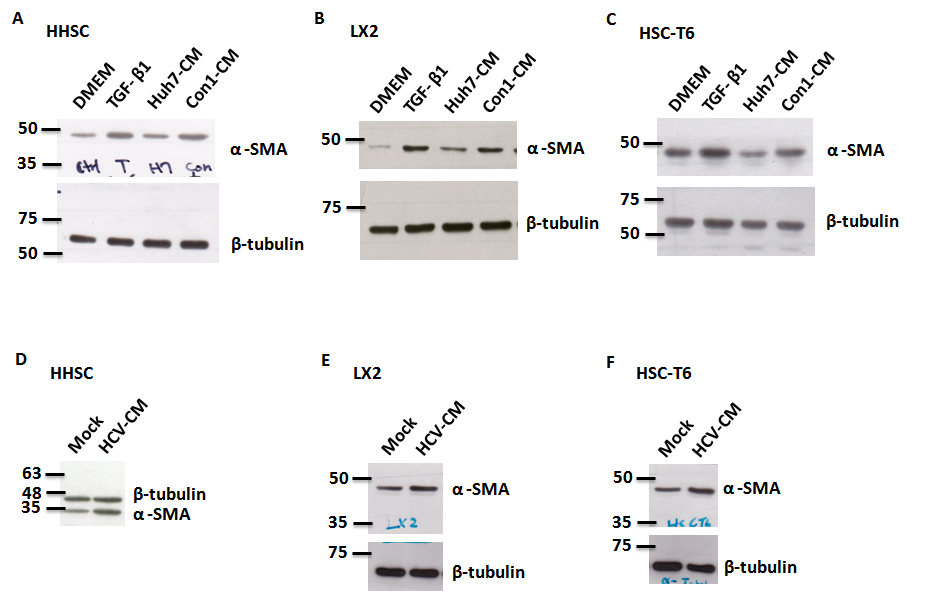
**

**Supplementary Fig. S1 Full Western blots of Fig. 1.**

**
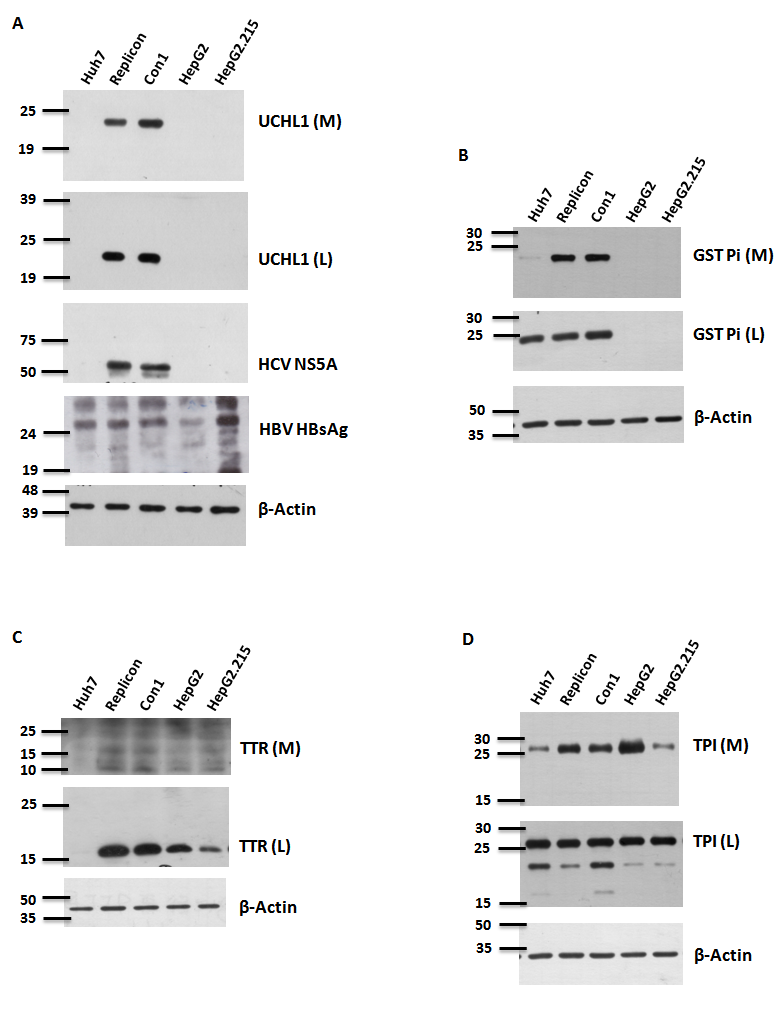
**

**Supplementary Fig. S2 Full Western blots of Fig. 3.**

**Fig. S2A: full Western blot of Fig. 3A .**

**Fig. S2B: full Western blot of Fig. 3B.**

**Fig. S2C: full Western blot of Fig. 3C.**

**Fig. S2D: full Western blot of Fig. 3D.**

**
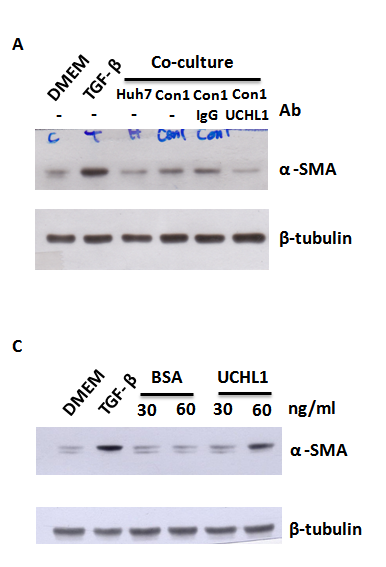
**

**Supplementary Fig. S3 Full Western blots of Fig. 5.**

**Fig. S3A: full Western blot of Fig. 5A .**

**Fig. S3C: full Western blot of Fig. 5C.**

**
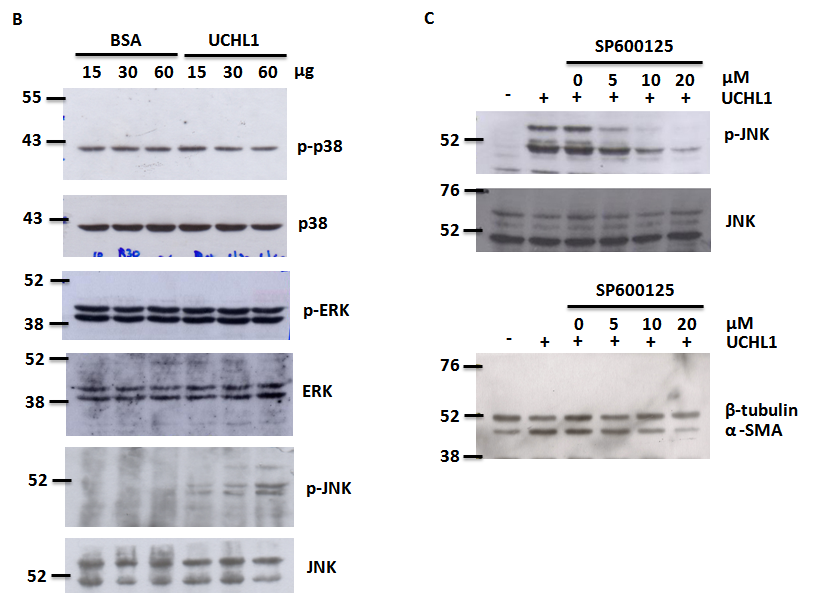
**

**Supplementary Fig. S4 Full Western blots of Fig. 6.**

**Fig. S4B: full Western blot of Fig. 6B.**

**Fig. S4C: full Western blot of Fig. 6C.**

**
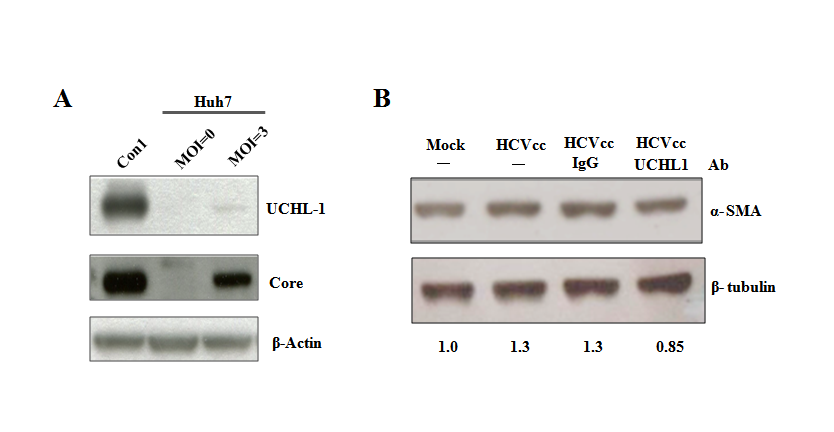
**

**Supplementary Fig. S5. The anti-UCHL1 neutralization antibody decreased the activation of HSCs by conditioned medium from the culture of HCVcc-infected cells.** (A) The Huh7 cells were infected with mock or HCVcc (JFH1, MOI 3) for 4 h and cultured for an additional 72 h after removing HCV infectious particles by washing three times with DMEM followed by suspension of the Huh7 cells in DMEM supplemented with 0.2% FBS. The cell lysates were collected to detect the expression of UCHL1 by Western blotting analysis. (B) The LX2 cells were culture in DMEM in the absence of FBS for 24 h and then treated with the conditioned medium derived from the 72 h culture of Huh7 cells infected with HCVcc for an additional 24 h. The cell lysates were harvested and Western blot analysis was performed using the anti α-SMA antibody. The ratios for the relative band intensities of α-SMA normalized by the expression of β-tubulin are shown at the bottom.

**
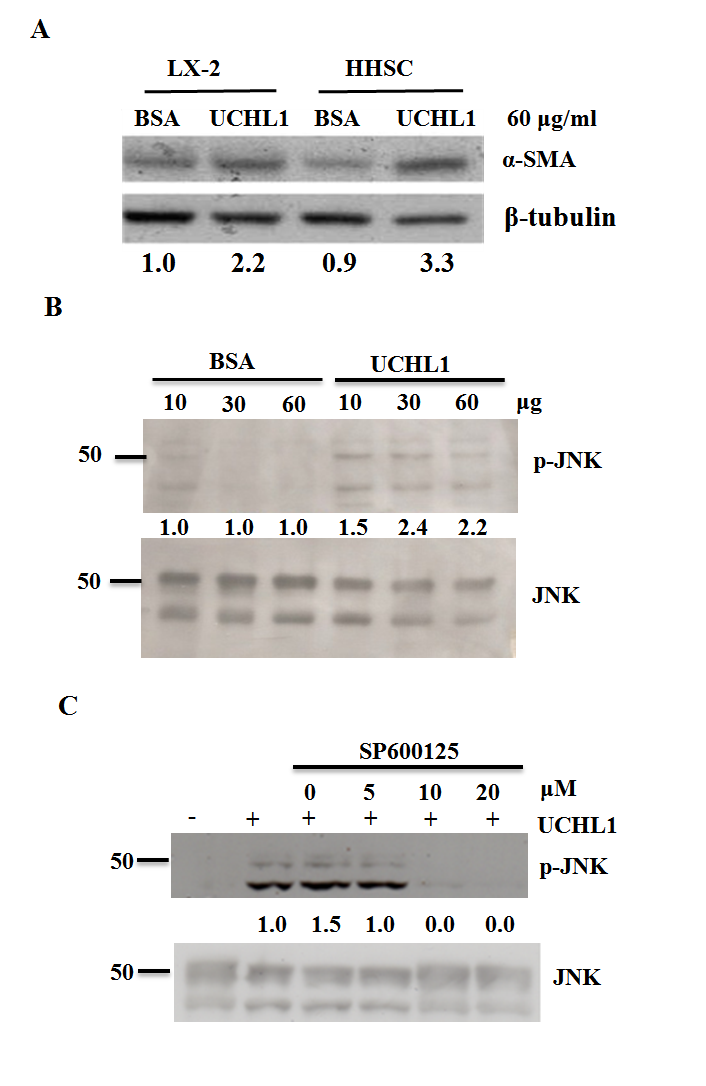
**

**Supplemental Fig. S6**. **UCHL1 induced LX2 activation through JNK phosphorylation.** (A) LX2 or HHSC cells were cultured in DMEM w/o FBS for 24 h and then treated with 60 μg of purified rUCHL1 or BSA for additional 24 h. The cell lysates were collected and the expression of α-SMA was determined by Western blot analysis. The expression of β-tubulin was used as the control for equal protein loading. The number below the gel images represents the relatively α-SMA expression levels for the indicated experimental condition after normalization with the expression of β-tubulin. (B) LX2 cells were treated with increasing concentrations of BSA or purified rUCHL1 protein for 24 h. The expression and the phosphorylation status of JNK were analyzed by Western blotting. (C) LX2 cells were treated with the indicated concentrations of JNK inhibitor SP600125 for 30 min in DMEM supplemented with 0.2% FBS. Purified rUCHL1 (60 μg) proteins were then added into the culture medium for 1 h to detect the expression and phosphorylation status of JNK by Western blotting. The number below the gel images represents the relatively phosphorylation status of JNK for the indicated experimental condition after normalization with the expression of JNK.
